# Persistent neuronal *Ube3a* expression in the suprachiasmatic nucleus of Angelman syndrome model mice

**DOI:** 10.1038/srep28238

**Published:** 2016-06-16

**Authors:** Kelly A. Jones, Ji Eun Han, Jason P. DeBruyne, Benjamin D. Philpot

**Affiliations:** 1 Department of Cell Biology & Physiology, UNC Neuroscience Center, University of North Carolina School of Medicine, Chapel Hill, NC 27599, USA; 2 Carolina Institute for Developmental Disabilities, University of North Carolina School of Medicine, Chapel Hill, NC 27599, USA; 3 Department of Pharmacology & Toxicology, Neuroscience Institute, Morehouse School of Medicine, Atlanta, GA 30310, USA

## Abstract

Mutations or deletions of the maternal allele of the *UBE3A* gene cause Angelman syndrome (AS), a severe neurodevelopmental disorder. The paternal *UBE3A/Ube3a* allele becomes epigenetically silenced in most neurons during postnatal development in humans and mice; hence, loss of the maternal allele largely eliminates neuronal expression of UBE3A protein. However, recent studies suggest that paternal *Ube3a* may escape silencing in certain neuron populations, allowing for persistent expression of paternal UBE3A protein. Here we extend evidence in AS model mice (*Ube3a*^*m*–/*p*+^) of paternal UBE3A expression within the suprachiasmatic nucleus (SCN), the master circadian pacemaker. Paternal UBE3A-positive cells in the SCN show partial colocalization with the neuropeptide arginine vasopressin (AVP) and clock proteins (PER2 and BMAL1), supporting that paternal UBE3A expression in the SCN is often of neuronal origin. Paternal UBE3A also partially colocalizes with a marker of neural progenitors, SOX2, implying that relaxed or incomplete imprinting of paternal *Ube3a* reflects an overall immature molecular phenotype. Our findings highlight the complexity of *Ube3a* imprinting in the brain and illuminate a subpopulation of SCN neurons as a focal point for future studies aimed at understanding the mechanisms of *Ube3a* imprinting.

Mutations or deletions of the maternal allele of *UBE3A* cause Angelman syndrome (AS), a rare neurodevelopmental disorder characterized by developmental delay, lack of speech, seizures, motor abnormalities, happy affect, and sleep disturbances^1^. The *UBE3A* gene exhibits preferential expression from the maternal allele in a majority of neuronal subclasses, while its expression appears to be biallelic in other cell types^2^,^3^. Neuronal imprinting of *UBE3A* is achieved through paternal expression of an antisense transcript, *UBE3A-ATS*, which extends across the *UBE3A* gene and suppresses its expression in *cis*^4^,^5^. An early study in mice showed that expression of *Ube3a* is nearly exclusively maternal in neurons within the CA3 region of the hippocampus and in cerebellar Purkinje cells, with moderate maternal bias in the cerebral cortex^6^. Later work using a UBE3A-YFP (yellow fluorescent protein) fusion reporter mouse line demonstrated that *Ube3a* expression is preferentially maternal in neurons throughout the cortex, hippocampus, cerebellum, and thalamus^7^. The maternal bias of UBE3A expression is established perinatally, and relaxed imprinting of *Ube3a* has been observed in early postnatal mouse visual cortex^8^ and cortical lysates^9^. Indeed, the paternal *Ube3a* allele is silenced in neurons in the mouse neocortex between birth and postnatal day 7 (P7), and at P7 granule cells in the dentate gyrus and cerebellum still exhibit paternal *Ube3a* expression^10^. These observations demonstrate a variable onset of imprinting across brain regions, likely related to differences in the timing of neuronal differentiation. While a clearer picture of parental *Ube3a* expression bias in many forebrain structures has begun to emerge, detailed knowledge of allelic contributions to *Ube3a* expression in the basal telencephalon, particularly the hypothalamus, is lacking.

Sleep disturbances in individuals with AS persist throughout childhood and manifest as reduced need for sleep, difficulties falling asleep, and sleep fragmentation^11^,^12^,^13^. Sleep disturbances in AS model mice (*Ube3a*^*m*–/*p*+^), in which a null allele of *Ube3a* is maternally inherited, have also been consistently observed^14^,^15^. Because circadian rhythms play a critical role in determining sleep onset, duration, and quality^16^, the possibility has been raised that disruptions in circadian rhythms might underlie the sleep disturbances observed in AS. UBE3A has been shown to interact with an important member of the molecular clock^17^,^18^, implicating it in the molecular mechanisms that drive circadian rhythmicity. We previously observed persistent expression of UBE3A in the suprachiasmatic nucleus (SCN) of the hypothalamus, the master circadian regulatory region in the mammalian brain, of AS model mice^15^, thus identifying a novel site for relaxation of maternal expression bias of *Ube3a* in the adult brain. Here we examine the expression patterns of UBE3A in the SCN of AS model mice and provide evidence for paternal expression of *Ube3a* in a subset of neurons in this circadian regulatory region. The persistence of paternal UBE3A may be important for SCN function and, together with other markers of young neurons, suggests a degree of neoteny in the molecular profile of adult SCN neurons.

## Results

### Characteristics of UBE3A immunofluorescence in the SCN of adult AS model mice

We previously made the surprising finding that UBE3A protein is expressed in the SCN of adult AS model mice^15^. To characterize the distribution of UBE3A expression throughout the SCN, we performed immunohistochemistry in coronal sections spanning the entire rostro-caudal extent of the SCN from wildtype (*Ube3a*^*m*+/*p*+^), AS model (*Ube3a*^*m*–/*p*+^), and homozygous null (*Ube3a*^*m*–/*p*–^) mice. For these experiments we used a previously characterized antibody^10^,^15^ and an immunofluorescence approach optimized for sensitive detection of UBE3A^10^.

**Figure 1:**
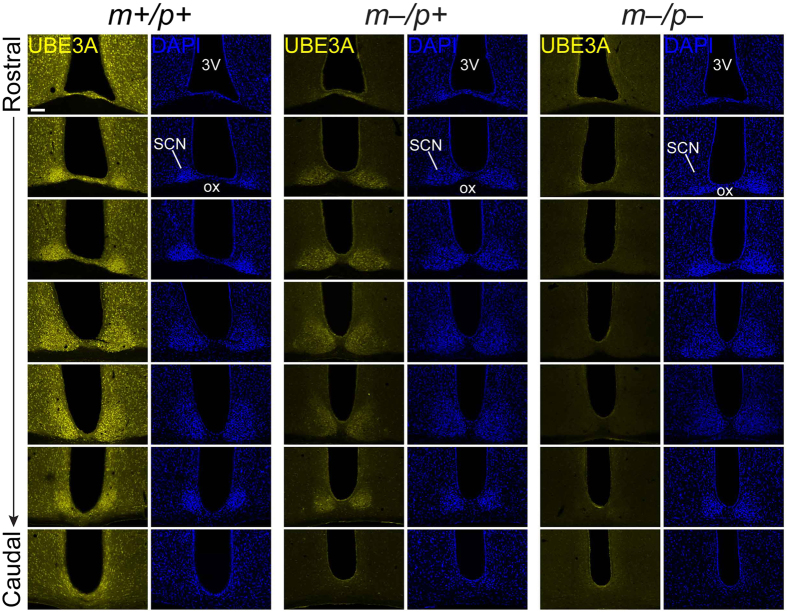
UBE3A expression in the SCN of wildtype and AS model mice along the rostral-caudal axis. Representative confocal images of UBE3A immunofluorescence and DAPI counterstaining in rostral-to-caudal series of coronal sections through the SCN of wildtype (*Ube3a*^*m*+/*p*+^), AS model (*Ube3a*^*m*–/*p*+^), and homozygous null (*Ube3a*^*m*–/*p*–^) mice. DAPI costaining demarcates SCN regions. UBE3A signal is detectable in *Ube3a*^*m*–/*p*+^ sections containing the SCN but not in hypothalamic regions outside of the SCN, in regions rostral or caudal to the SCN (topmost or bottommost panels), or in sections from *Ube3a*^*m*–/*p*–^ mice. *3V*, third ventricle; *ox*, optic chiasm. Scale bar, 100 μm.

**Figure 2:**
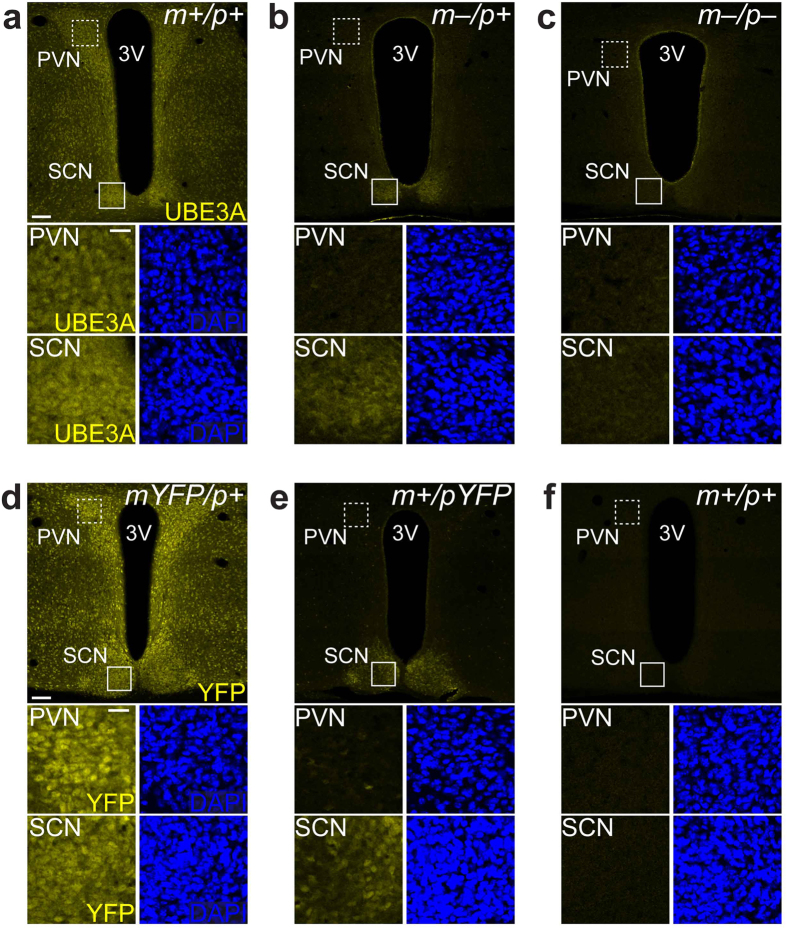
Paternal UBE3A expression in the SCN but not the PVN in AS model mice. (**a**–**c**) Confocal micrographs of UBE3A immunofluorescence in hypothalamic regions surrounding the third ventricle in *Ube3a*^*m*+/*p*+^ (**a**), *Ube3a*^*m*–/*p*+^ (**b**), and *Ube3a*^*m*–/*p*–^ (**c**) mice. DAPI counterstaining demarcates SCN and PVN. High-magnification images show that UBE3A signal is observable in the SCN (solid boxes) and PVN (dashed boxes) of *Ube3a*^*m*+/*p*+^ mice, as well as the SCN, but not the PVN, of *Ube3a*^*m*–/*p*+^ mice. UBE3A signal is absent in the SCN and PVN of *Ube3a*^*m*–/*p*–^ mice (**c**) as expected. (**d**–**f**) YFP immunofluorescence and DAPI counterstaining in hypothalamic regions surrounding the third ventricle in *Ube3a*^*mYFP*/*p*+^ (**d**), *Ube3a*^*m*+/*pYFP*^ (**e**), and *Ube3a*^*m*+/*p*+^ (**f**) mice. High-magnification images of SCN (solid boxes) and PVN (dashed boxes) show that YFP signal is present in both the SCN and PVN of *Ube3a*^*mYFP*/*p*+^ mice (**d**), but only in the SCN of *Ube3a*^*m*+/*pYFP*^ mice (**e**). YFP signal is absent in *Ube3a*^*m*+/*p*+^ mice (**f**). *3V*, third ventricle. Scale bars, 100 μm (**a**,**d**, top); 25 μm (**a,d**, bottom).

We observed strong UBE3A signal throughout the hypothalamus in wildtype mice that was absent in AS model mice, indicative of the specificity of the antibody (Fig. 1) and consistent with observations that the *Ube3a* mutant allele does not produce a detectable transcript or protein^19^. Notably, there was a subset of UBE3A-positive cells throughout the rostro-caudal extent of the SCN in AS model mice (Fig. 1), indicating persistent paternal *Ube3a* expression. This pattern was specific to the SCN, as regions immediately rostral or caudal to the SCN did not exhibit UBE3A that was readily apparent at this magnification (Fig. 1, topmost and bottommost panels). Moreover, while UBE3A expression patterns in the SCN and the nearby paraventricular nucleus (PVN) were comparable in wildtype mice (Fig. 2a), there was little UBE3A signal in the PVN compared to the SCN in AS model mice (Fig. 2b) and no detectable signal in homozygous null mice (Fig. 2c). Thus, paternal *Ube3a* expression is readily apparent in the SCN of AS model mice, but not in a similarly neuron-dense hypothalamic region.

To provide additional evidence of persistent paternal *Ube3a* expression in the SCN, we employed a transgenic mouse line expressing a knockin allele in which yellow fluorescent protein (YFP) is fused to the carboxyl-terminus of UBE3A^7^. Maternal inheritance of this reporter allele (*Ube3a*^*mYFP*/*p*+^) yielded UBE3A-YFP expression in virtually all neurons in the SCN and surrounding hypothalamus, including the PVN (Fig. 2d). Mirroring patterns of endogenous paternal UBE3A signal (Figs 1 and 2a–c), UBE3A-YFP expression in *Ube3a*^*m*+/*pYFP*^ mice was present in a subset of cells in the SCN, but paternal UBE3A-YFP expression was not detectable in surrounding hypothalamic regions (Fig. 2e). As expected, UBE3A-YFP expression was absent in control littermate mice lacking the reporter allele (*Ube3a*^*m*+/*p*+^) (Fig. 2f). Notably, while UBE3A-YFP signal in the SCN and PVN of *Ube3a*^*mYFP*/*p*+^ mice was comparable, UBE3A-YFP signal in the PVN of *Ube3a*^*m*+/*pYFP*^ mice was dramatically reduced compared to the SCN (Fig. 2e). These data indicate that cells in the SCN express paternal UBE3A, while neurons in the PVN do not, and further confirm that the UBE3A immunofluorescent signal in the SCN of AS model mice reflects expression from the paternal allele.

### Paternal UBE3A in the SCN colocalizes with SOX2, a marker of neuronal immaturity

**Figure 3:**
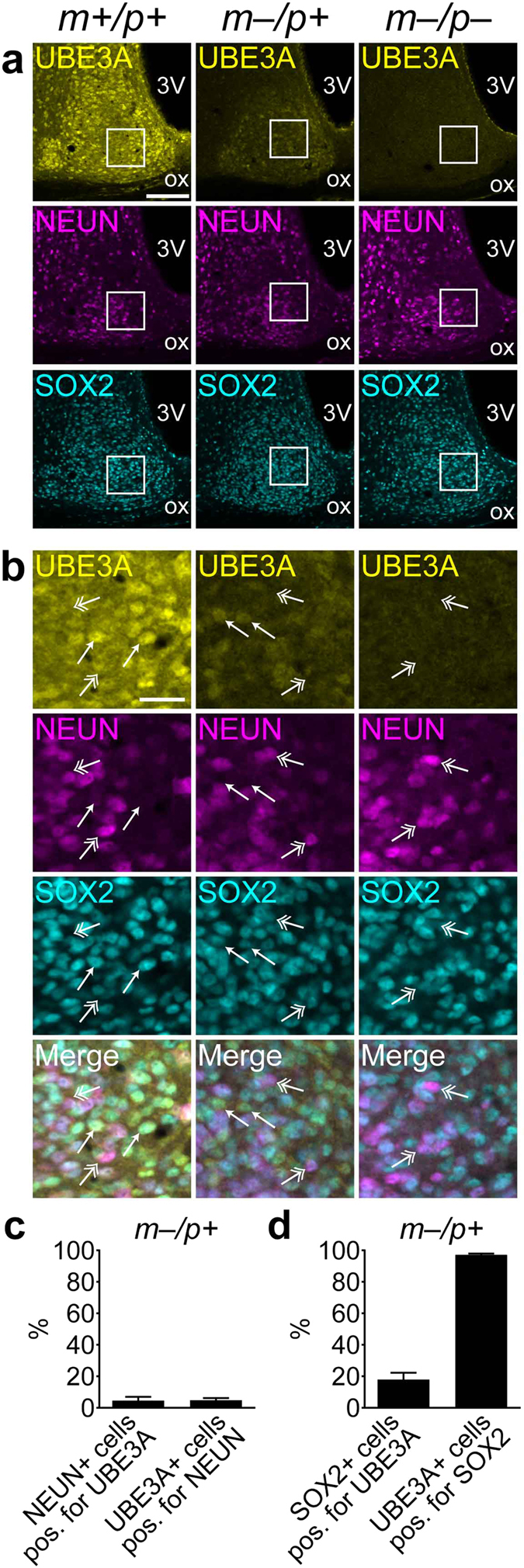
Paternal UBE3A and SOX2 primarily colocalize in NEUN-negative cells in the SCN of AS model mice. (**a**,**b**) Representative confocal images of UBE3A, SOX2, and NEUN immunofluorescent signals in the SCN of *Ube3a*^*m*+/*p*+^, *Ube3a*^*m*–/*p*+^ and *Ube3a*^*m*–/*p*–^ mice. (**b**) High-magnification images of SCN regions in solid white boxes shown in **a**. UBE3A and SOX2 colocalize in many cells (arrows), which are largely distinct from NEUN-positive cells (double-headed arrows). (**c**) Quantification of colocalization between UBE3A and NEUN in the SCN of *Ube3a*^*m*–/*p*+^ mice (UBE3A-positive cells positive for NEUN, n = 99–282 cells per section, 3 sections each from 3 *Ube3a*^*m*–/*p*+^ mice; NEUN-positive neurons positive for UBE3A, n = 125–376 cells per section, 3 sections each from 3 *Ube3a*^*m*–/*p*+^ mice). (**d**) Quantification of colocalization between UBE3A and SOX2 in the SCN of *Ube3a*^*m*–/*p*+^ mice (SOX2-positive cells positive for UBE3A, n = 386–1308 cells from 3 sections each from 4 *Ube3a*^*m*–/*p*+^ mice; UBE3A-positive cells positive for SOX2, n = 77–263 cells from 3 sections each from 4 *Ube3a*^*m*–/*p*+^ mice). *3 V*, third ventricle; *ox*, optic chiasm. Scale bars, 100 μm (**a**); 25 μm (**b**).

Paternal *Ube3a* expression is a characteristic of glial cells, young or newly differentiated neurons, and postnatal neural stem cells^10^. Because many paternal UBE3A-positive cells in the SCN were large in diameter and lacked colocalization with the astrocytic marker GFAP (Supplementary Fig. S1), we hypothesized that they were neurons. To profile the relative maturity of putative neurons expressing paternal UBE3A in the SCN, we visualized UBE3A and NEUN immunofluorescent signals in the SCN of adult wildtype, AS model, and homozygous-null mice, along with SOX2 (Fig. 3a,b). NEUN is a marker of mature neurons^20^,^21^; however, consistent with previous observations^22^,^23^,^24^, distribution of NEUN immunofluorescence was heterogeneous in the SCN, with stronger expression in a central region and minimal expression in dorsal and medial regions (Fig. 3a,b). Paternal UBE3A-positive cells in AS model mice were largely negative for NEUN, as measured by cell counting (4.8% ± 0.76) (Fig. 3c). Similarly, only a small proportion of NEUN-positive neurons were also positive for paternal UBE3A (4.6% ± 1.4) (Fig. 3c). Higher resolution imaging confirmed that most cells positive for either endogenous paternal UBE3A or paternal UBE3A-YFP did not express NEUN (Supplementary Fig. S2). These findings are concordant with previous work showing an inverse relationship between paternal *Ube3a* expression and NEUN expression during postnatal neuronal development in the neocortex^10^. To provide complementary evidence for neuronal paternal UBE3A expression, we co-stained sections containing the SCN with NeuroTrace Fluorescent Nissl stain (Supplementary Fig. S3). Nissl staining could be observed throughout the SCN, while only a subset of Nissl-stained cells expressed NEUN in the SCN, as previously observed^23^. UBE3A immunofluorescence was localized to Nissl-stained cells, in SCN sections from both wildtype and AS model mice. In sections from AS model mice, 90.1 ± 0.776% of UBE3A-positive cells were also positive for Nissl stain (n = 3 mice, 2–3 sections per mouse, 88–176 cells per section).

We also employed other markers to determine the neuronal identity of paternal UBE3A-expressing cells. One such marker, *SOX2* (sex-determining region Y [SRY]-related HMG box 2), is a member of the *SOXB1* family of transcription factors and plays important roles in maintaining neural progenitors during development and in adult neural stem cell niches^25^,^26^. Surprisingly, *Sox2* expression has been observed in neurons of the adult rodent SCN but not in other hypothalamic regions^27^, an unusual finding given *Sox2*’s role in maintaining neural stem cell populations. We hypothesized that SCN neurons expressing paternal UBE3A might also express SOX2, as both markers are strongly associated with neuronal immaturity. In contrast to NEUN, SOX2 expression was widespread across the SCN in all genotypes, similar to the pattern previously described^27^, and exhibited substantial colocalization with UBE3A in the SCN of wildtype and AS model mice (Fig. 3a,b). In the SCN of AS model mice, nearly all paternal UBE3A-positive cells were also positive for SOX2 (97.2% ± 0.351), whereas a minority of SOX2-positive neurons were also positive for paternal UBE3A (18% ± 2.2) (Fig. 3d). As previously observed^27^, NEUN and SOX2 expression only partially overlapped, which is most apparent in the merged representative image for the homozygous null condition (Fig. 3b). Thus, in addition to SOX2 and perhaps other proteins, paternal UBE3A comprises the molecular profile of immature neurons in the adult SCN.

### Many neurons expressing paternal Ube3a in the SCN of AS model mice are AVP-positive

**Figure 4:**
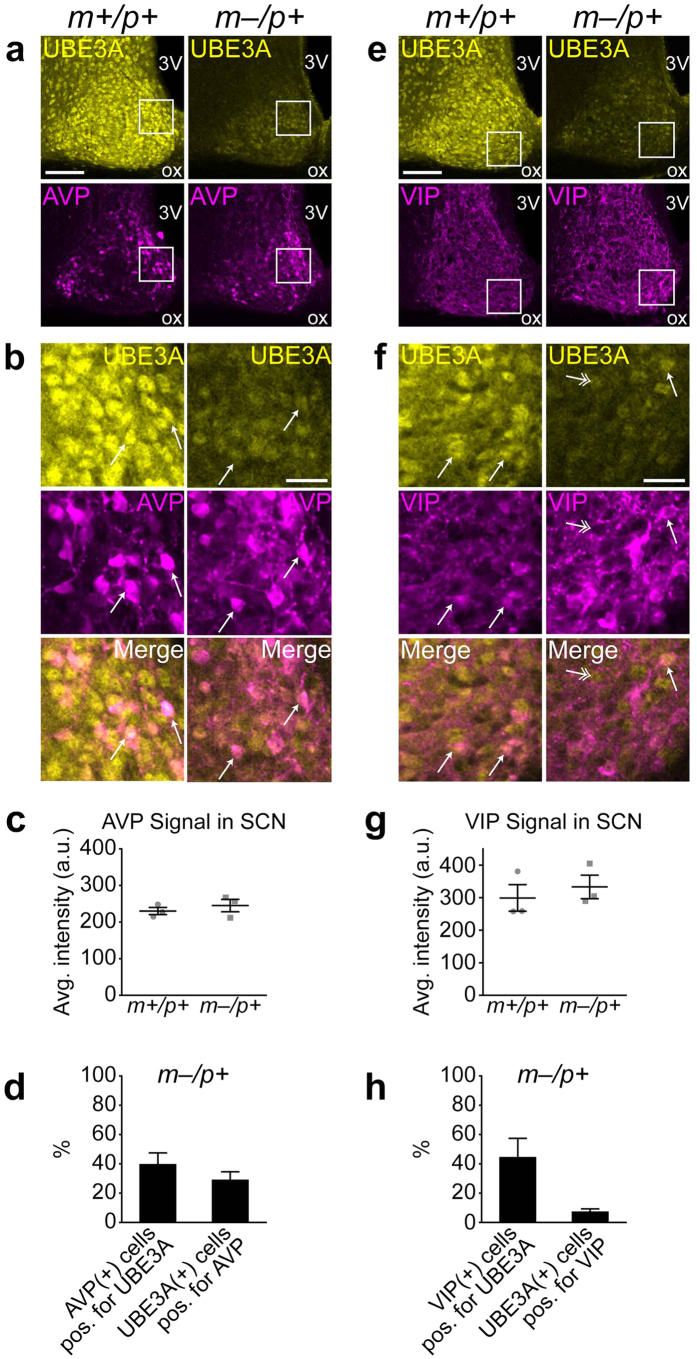
Colocalization of UBE3A with AVP and VIP in the SCN of AS model mice. (**a**) Representative confocal images of immunofluorescent signals of UBE3A and AVP in coronal sections containing the SCN from *Ube3a*^*m*+/*p*+^ and *Ube3a*^*m*–/*p*+^ mice sacrificed at ZT8 on a 12:12 LD cycle. (**b**) High-magnification images of regions in white boxes in **a**. Arrows identify cells co-expressing UBE3A and AVP. (**c**) Quantification of average intensity of AVP immunofluorescence in SCN regions in *Ube3a*^*m*+*/p*+^ and *Ube3a*^*m*–/*p*+^ mice (p = 0.484, Student’s *t* test, n = 3 mice). (**d**) Quantification of AVP-UBE3A colocalization in the SCN of *Ube3a*^*m*–/*p*+^mice (AVP-positive neurons positive for UBE3A, n = 28–149 cells from 3 sections each from 3 mice; UBE3A-positive neurons positive for AVP: n = 44–274 cells from 3 sections each from 3 mice). (**e**) Representative images of UBE3A and VIP immunofluorescent signals in coronal sections containing the SCN from *Ube3a*^*m*+*/p*+^ and *Ube3a*^*m*–/*p*+^ mice sacrificed at ZT4 on a 12:12 LD cycle. (**f**) High-magnification images of region in white boxes in **e**. Arrows identify cells co-expressing UBE3A and VIP; double-headed arrows indicate UBE3A-positive cells that lack VIP expression. (**g**) Quantification of average intensity of VIP immunofluorescence in SCN regions in *Ube3a*^*m*+*/p*+^ and *Ube3a*^*m*–/*p*+^ mice (p = 0.568, Student’s *t* test, n = 3 mice). (**h**) Quantification of VIP-UBE3A colocalization in the SCN of *Ube3a*^*m*–/*p*+^ mice (VIP-positive neurons positive for UBE3A: n = 4–56 cells from 1–3 sections each from 5 mice; UBE3A-positive neurons positive for VIP: n = 50–243 cells from 1–3 sections each from 5 mice). Scale bars, 100 μm (**a**, **e**), 25 μm (**b**,**f**).

We next asked whether paternal UBE3A expression would overlap with one or more defined neuron populations in the SCN. Neuropeptide signaling is a key mechanism mediating intercellular communication among heterogeneous cell populations in the SCN neural circuit^28^. The neuropeptide arginine vasopressin (AVP) is synthesized and released from neurons in the SCN “shell”, which corresponds generally to the dorsomedial subdivision of the SCN^29^. We analyzed expression levels and the degree of colocalization of UBE3A and AVP in the SCN of wildtype and AS model mice sacrificed at *zeitgeber* time (ZT) 8 on a 12:12 light/dark (LD) cycle (Fig. 4a,b). Average intensity of AVP immunofluorescent signals across the SCN were not different between wildtype and AS model mice (wildtype, 230 ± 9.65 arbitrary units (a.u.); AS model, 245 ± 17.0 a.u.; p = 0.484, Student’s *t* test, n = 3) (Fig. 4c), illustrating that loss of maternal UBE3A does not impact AVP expression, either in cell bodies or neuronal processes. In the SCN of AS model mice, a subpopulation of AVP-positive neurons were positive for UBE3A (40% ± 4.4), as measured by cell counting, and a subpopulation of paternal UBE3A-positive neurons were also positive for AVP (29% ± 3.1) (Fig. 4d). While overlap with AVP-expressing neurons was moderate, paternal UBE3A expression was also present in cells outside of the dorsomedial regions of the SCN, as shown in lower-magnification images (Fig. 4a). These data suggest that paternal *Ube3a* expression in the SCN of AS model mice is not restricted to AVP-expressing neurons or the dorsomedial SCN.

Vasoactive intestinal peptide (VIP) is a neuropeptide that is critically important in the SCN for intercellular communication from light-responsive “core” neurons to shell neurons, which receive minimal retinal input^30^. We examined VIP expression levels and colocalization with paternal UBE3A in the SCN of AS model mice (Fig. 4e,f). The average intensity of VIP immunofluorescent signal in the SCN was not different between wildtype and AS model mice sacrificed at ZT4 (wildtype, 299 ± 40.8 a.u.; AS model, 333 ± 36.2 a.u.; p = 0.568, Student’s *t* test, n = 3 mice) (Fig. 4g). While some VIP neurons were positive for paternal UBE3A (45% ± 5.7), only a small number of paternal UBE3A-positive neurons were also positive for VIP (7.5% ± 0.79) (Fig. 4h). Furthermore, cells expressing paternal UBE3A were found throughout the SCN beyond the ventral regions where VIP neurons are enriched (Fig. 4e).

Taken together, these findings show that loss of maternal *Ube3a* does not disrupt expression levels of either AVP or VIP across the SCN. Furthermore, our results reveal that UBE3A-positive neurons in AS model mice are more likely to express AVP than VIP in the SCN, but paternal *Ube3a* expression was not restricted to either of these SCN populations.

### Paternal UBE3A-positive cells in the SCN express PER2 and BMAL1

**Figure 5:**
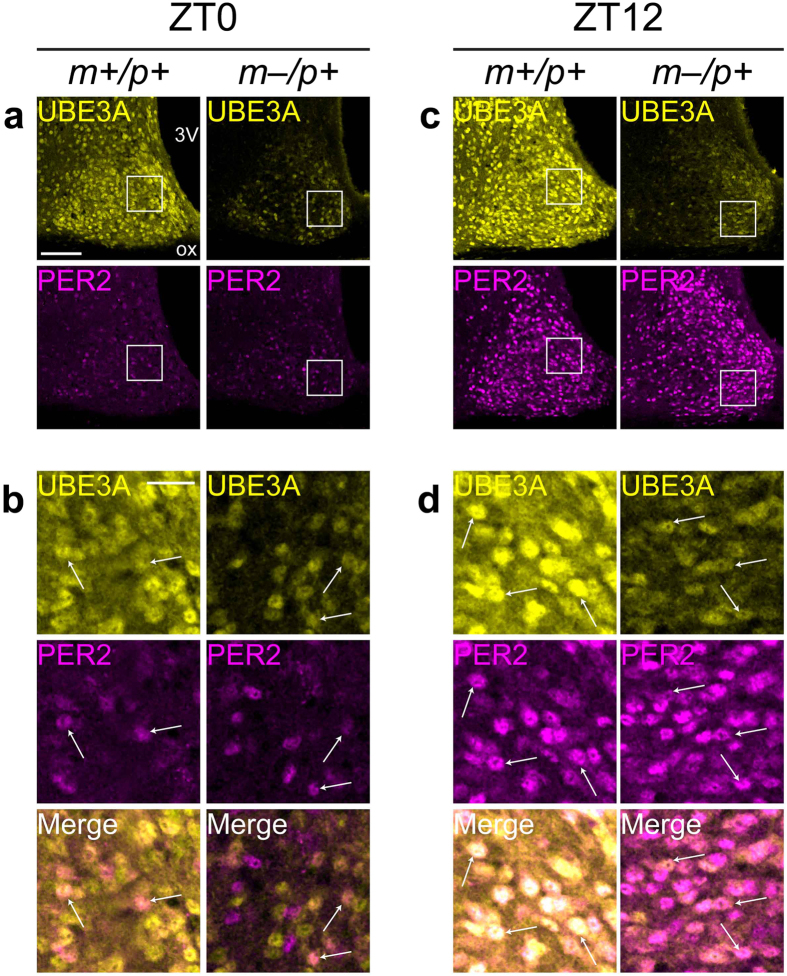
Paternal UBE3A is expressed in PER2-positive neurons in the SCN. (**a**–**d**) Representative confocal micrographs of UBE3A and PER2 immunofluorescent signals in the SCN of *Ube3a*^*m*+*/p*+^ and *Ube3a*^*m*–/*p*+^ mice sacrificed at ZT0 (**a**,**b**) or ZT12 (**c**,**d**) on a 12:12 LD cycle. (**b,d**) High-magnification images of regions in white boxes in **a** and **c** respectively, showing colocalization of UBE3A with PER2 (arrows in **b** and **d**). Scale bars, 100 μm (**a**,**c**), 25 μm (**b**,**d**).

Period homolog 2 (*PER2*) is a key component of the transcriptional-translational feedback loops that drive circadian rhythms in the SCN^31^ and exhibits strong 24-hr expression rhythms in SCN neurons^32^. A recent study revealed lengthened circadian period using SCN tissue explants from AS model mice expressing the P_mPer2_::mPER2-LUC reporter^17^; however, we recently showed that rhythms in PER2 protein expression were not different between wildtype and AS model mice across a 12:12 light/dark cycle^15^. To address whether paternal UBE3A-positive neurons express PER2 and exhibit molecular rhythms, we examined colocalization of UBE3A and PER2 immunofluorescent signals in the SCN of wildtype and AS model mice sacrificed either at ZT0 (corresponding to lights-on, when PER2 levels are lowest) or ZT12 (lights-off, when PER2 levels are highest). AT ZT0, when PER2 expression was sparse, a subset of PER2-positive neurons also expressed paternal UBE3A in AS model mice (Fig. 5a,b). At ZT12, when PER2 expression was widespread, most UBE3A-positive cells in the SCN of AS model mice strongly expressed PER2 (Fig. 5c,d). PER2 immunofluorescent signals were not different between genotypes at either ZT0 or ZT12, as measured previously^15^. Thus, paternal *Ube3a* expression occurs in SCN neurons that participate in circadian timing.

**Figure 6:**
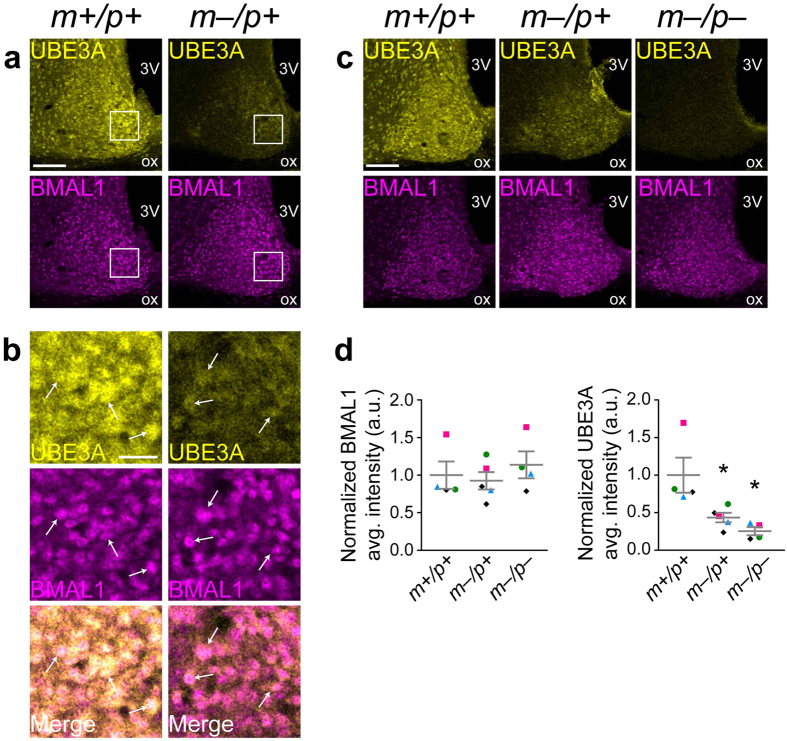
Paternal UBE3A is expressed in BMAL1-positive neurons in the SCN. (**a**,**b**) Representative confocal micrographs of UBE3A and BMAL1 immunofluorescent signals in the SCN of *Ube3a*^*m*+*/p*+^ and *Ube3a*^*m*–/*p*+^ mice sacrificed at ZT0 (lights-on) on a 12:12 LD cycle. (**b**) High-magnification images of regions in white boxes in **a**. UBE3A-positive cells in sections from *Ube3a*^*m*–/*p*+^ mice also express BMAL1 (arrows in **b**). (**c**,**d**) BMAL1 immunofluorescence is similar in the SCN of *Ube3a*^*m*+*/p+*^, *Ube3a*^*m*–/*p*+^, and *Ube3a*^*m*–/*p*–^ mice (one-way ANOVA, F_(2,10)_ = 0.4777, p = 0.6337, n = 4–5 mice). UBE3A immunofluorescence is significantly decreased in *Ube3a*^*m*–/*p*+^and *Ube3a*^*m*–/*p*–^ mice (one-way ANOVA, F_(2,10)_ = 7.982, p = 0.0085, n = 4–5 mice; *p < 0.05, **p < 0.01 compared to *Ube3a*^*m*+*/p*+^). Symbols indicate mice of each genotype that were processed together and compared within each experiment. Representative images in **c** correspond to black diamonds in graphs (2 *Ube3a*^*m*–/*p*+^ mice from that cohort were included for quantification, and the representative *Ube3a*^*m*–/*p*+^ image corresponds to the points with higher BMAL1 and UBE3A intensity values in each graph). Scale bars, 100 μm (**a**,**c**), 25 μm (**b**).

*BMAL1* is another central component of the transcriptional-translational feedback loops that comprise the molecular clock^31^. Recent work has implicated UBE3A in the regulation of BMAL1 stability. Activation of UBE3A in heterologous cells, induced by overexpression of the viral oncogenes E6/E7, increased ubiquitination of BMAL1, and UBE3A can interact with, ubiquitinate, and degrade BMAL1^18^. A subsequent study showed that UBE3A interacts with BMAL1 in mouse brain, and that BMAL1 protein levels in the hypothalamus as detected by immunoblot were increased in AS mouse models compared with wildtype controls, providing evidence for a role for UBE3A in degrading BMAL1 in the brain^17^. We therefore examined whether paternal UBE3A expression overlapped with BMAL1 expression in the SCN (Fig. 6). BMAL1 immunofluorescence in the SCN at ZT0 was high in the majority of neurons in both wildtype and AS model mice (Fig. 6a). Paternal UBE3A expression coincided with BMAL1 expression to a high degree; we rarely observed cells clearly expressing either protein alone (Fig. 6b). We also quantified the BMAL1 immunofluorescent signal in the SCN of wildtype, AS model, and homozygous-null mice sacrificed at ZT8 (a time when effects on BMAL1 stability should be readily apparent due to low mRNA expression), and found no significant differences among genotypes (one-way ANOVA, F_(2,10)_ = 0.4777, p = 0.6337, n = 4–5 mice) (Fig. 6c,d). Furthermore, when we examined BMAL1 levels across the day in the SCN of wildtype and AS model mice, we saw no difference in BMAL1 levels between genotypes at any time examined (Supplementary Fig. S4). However, Shi and colleagues reported that BMAL1 protein abundance in immunoblots of hypothalamus extracts were elevated in *Ube3a*^*m*–/*p*+^ mice^17^. This apparent discrepancy suggests that hypothalamic BMAL1 levels, outside the SCN, may be more drastically affected by UBE3A loss, perhaps directly related to the persistent UBE3A only within the SCN. Taken together, our data show that paternal UBE3A-positive neurons in the SCN express BMAL1, and that maternal or even complete loss of *Ube3a* does not detectably perturb BMAL1 levels in the SCN.

### Paternal Ube3a expression in the extended amygdala

**Figure 7:**
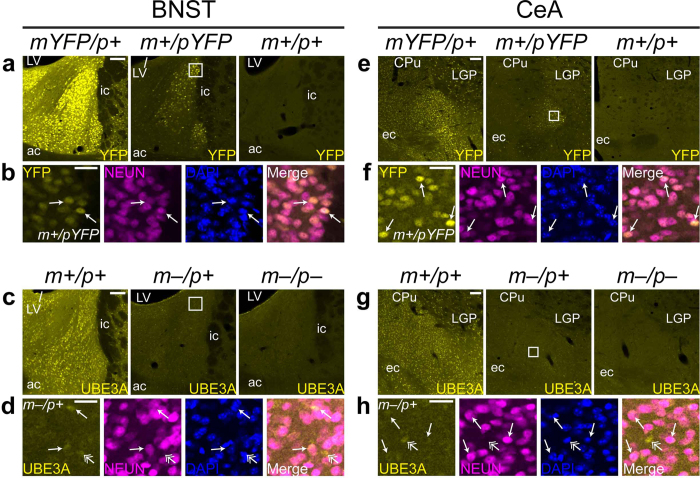
Paternal UBE3A expression in a subset of cells in the extended amygdala of AS model mice. (**a**–**d**) UBE3A expression in the BNST in *Ube3a*^*mYFP/p*+^, *Ube3a*^*m*+*/pYFP*^, and *Ube3a*^*m*+*/p*+^ mice (**a**,**b**), and *Ube3a*^*m*+*/p*+^, *Ube3a*^*m*–/*p*+^, and *Ube3a*^*m*–/*p*–^ mice (**c**,**d**). In **a**, images encompassing the BNST from *Ube3a*^*m*+*/pYFP*^ and *Ube3a*^*m*+*/p*+^ mice were adjusted for brightness and contrast independently from the *Ube3a*^*mYFP/p*+^ image to make the background staining appreciable. (**b**,**d**) High-magnification images of paternal UBE3A-YFP (**b**) or paternal UBE3A (**d**) in boxed region in **a** and **c**, respectively. Arrows indicate cells co-expressing paternal UBE3A and NEUN; double-headed arrows in **d** indicate cells expressing UBE3A but not NEUN. (**e**–**h**) UBE3A expression in the CeA in sections from *Ube3a*^*mYFP/p*+^, *Ube3a*^*m*+*/pYFP*^, and *Ube3a*^*m+/p*+^ mice (**e**,**f**), and *Ube3a*^*m*+*/p*+^, *Ube3a*^*m*–/*p*+^, and *Ube3a*^*m*–/*p*–^ mice (**g**,**h**). (**f**,**h**) High-magnification images of paternal UBE3A-YFP (**f**) or paternal UBE3A (**h**) in boxed region in **e** and **g**, respectively. Arrows indicate cells co-expressing paternal UBE3A and NEUN; double-headed arrows in **h** indicate cells expressing UBE3A but not NEUN. “Merge” images in **d** and **h** are overlays of UBE3A and NEUN channels. *LV*, lateral ventricle; *ac*, anterior commissure; *ic*, internal capsule; *CPu*, caudate putamen; *LGP*, lateral globus pallidus; *ec*, external capsule. Scale bars, 100 μm (**a**,**c**,**e**,**g**), 25 μm (**b**,**d**,**f**,**h**).

SCN-dependent rhythms in PER2 protein expression have been observed in the bed nucleus of the stria terminalis (BNST)^33^ and the central nucleus of the amygdala (CeA)^34^, regions that are part of the extended amygdala, a limbic macrostructure of the forebrain. We wondered whether paternal *Ube3a* expression might be a more general feature of neurons that undergo rhythmic changes in gene expression in the adult brain. We examined paternal *Ube3a* expression in the BNST and CeA of adult UBE3A-YFP-expressing and AS model mice (Fig. 7). UBE3A-YFP expression was abundant in the BNST of *Ube3a*^*mYFP*/*p*+^ mice, but not wildtype mice, as expected (Fig. 7a). Surprisingly, paternal UBE3A-YFP expression was detectable in a subset of BNST cells in *Ube3a*^*m*+/*pYFP*^ mice (Fig. 7a,b). Paternal UBE3A-YFP signal overlapped consistently with NEUN immunostaining (Fig. 7b), confirming that cells expressing paternal UBE3A-YFP in the adult BNST are mature neurons and demonstrating that paternal UBE3A can be expressed in neurons containing high levels of NEUN. Paternal expression of endogenous UBE3A in the BNST of AS model mice was qualitatively weaker than paternal UBE3A-YFP expression (compare Fig. 7a–d); however, paternal UBE3A signal was detectable in a subset of cells that co-expressed NEUN (Fig. 7d). In a similar fashion, paternal UBE3A-YFP was also expressed in a subset of NEUN-positive cells in the CeA, above the background fluorescence observed in wildtype sections (Fig. 7e,f), whereas paternal expression of endogenous UBE3A in CeA was much weaker (compare Fig. 7e–h), present in a minor subset of cells that co-expressed NEUN (Fig. 7h). Thus, relaxation of *Ube3a* imprinting extends to more regions of the adult brain than previously appreciated and may be a common feature of neurons with pronounced circadian rhythmicity of gene expression.

## Discussion

In this study, we have established that paternal *Ube3a* expression occurs in neurons throughout the SCN in adult mice. Neurons expressing paternal *Ube3a* were also likely to express SOX2, a marker of newly differentiated neurons, and were less likely to express the mature neuronal marker NEUN. Despite having molecular characteristics of immature neurons, paternal UBE3A-expressing neurons showed a time-of-day dependent change in PER2 levels, suggesting that they were functionally integrated into SCN circuitry. The BNST and CeA, regions of the extended amygdala that exhibit strong circadian rhythms of clock gene expression and control motivational behaviors, also exhibited paternal *Ube3a* expression in the adult. Our findings provide additional evidence for the persistence of markers of neuronal immaturity in the adult SCN, and highlight discrete groups of basal forebrain neurons in which paternal *Ube3a* appears to “escape” imprinting during postnatal development – knowledge that could be exploited to further elucidate molecular mechanisms of paternal *Ube3a* silencing.

We have provided evidence for the neuronal identity of paternal UBE3A-positive cells in the SCN. Paternal UBE3A expression exhibited substantial overlap with AVP, PER2, and BMAL1, proteins that are abundantly expressed in SCN neurons and are essential for SCN function and circadian rhythmicity. Due to its incomplete expression pattern in the SCN, the canonical neuronal marker NEUN was largely uninformative in defining the neuronal identity for paternal UBE3A-positive cells. Indeed, NEUN was expressed in SCN cells to the exclusion of paternal UBE3A and vice versa. Because previous observations suggest an inverse relationship between paternal UBE3A and NEUN expression in maturing neocortical neurons^10^, we speculate that regulation of these two genes is often coordinated.

It is possible that paternal UBE3A immunofluorescent signal observed in the SCN of AS model mice could be partially mediated by paternal UBE3A expression in glia, which biallelically express *Ube3a*^10^,^35^. Glia, particularly astrocytes, are likely to play a variety of roles in SCN function, including the support of rhythmic changes in metabolism and intrinsic excitability of SCN neurons^36^. Immunofluorescence of the astrocytic marker GFAP exhibits circadian-dependent changes in the SCN^37^, and astrocytes exhibit intrinsic clock rhythmicity^38^. The ratio of astrocytes to neurons in the SCN is approximately 1:3, based on classifications of nuclei morphologies using electron microscopy^39^. In our study, GFAP immunofluorescence illustrated that only a minority of cells expressing paternal UBE3A are astrocytes; for example, in a digital zoom of a given region of the SCN accounts for only 3–4 cells, while paternal UBE3A is expressed in >10 cells in the same region (Supplementary Fig. S1). Thus the observed number of cells expressing detectable paternal UBE3A is higher than could be accounted for by astrocytes. Taken together with the co-expression of neuronal proteins such as PER2, BMAL1, and AVP, our analysis points to neurons as the major cell class contributing to the observed pattern of paternal UBE3A expression.

The persistence of paternal *Ube3a* expression, previously ascribed to immature neurons, constitutes further evidence of an immature molecular phenotype in adult SCN neurons. In addition to minimal NEUN expression^23^, SCN neurons express a number of genes generally thought to be important only during development. We observed a high density of SOX2-positive cells in the SCN, similar to a pattern previously described^27^, and nearly all paternal UBE3A-positive cells were positive for SOX2. Moreover, SOX3, a cofactor of SOX2, is also expressed in adult SCN, in addition to neurogenic niches such as the subgranular zone of the hippocampal dentate gyrus^40^. Doublecortin-like, a microtubule-associated protein critical for embryonic neurogenesis, is expressed in NEUN-negative SCN shell neurons, but not in the PVN or the surrounding hypothalamus^24^. Doublecortin-like is involved in cellular migration and structural reorganization, and thus its enrichment in the SCN may enable the high degree of plasticity that is thought to be required to support circadian rhythms^41^,^42^. Thus, convergent evidence suggests a degree of neoteny in the molecular profile of SCN neurons, which may have important implications for SCN circuit development and function.

Paternal UBE3A-positive neurons in the adult SCN bear a molecular resemblance to newly generated neurons, which have yet to integrate into surrounding circuitry. Although the details of adult neurogenesis in the SCN specifically are not well understood, neurogenesis occurs at constitutive but low levels in the adult mouse hypothalamus^43^,^44^,^45^. However, paternal UBE3A is co-expressed by rhythmically PER2-expressing SCN neurons (Fig. 5), indicating that these neurons are not newly born, but rather are fully integrated into the surrounding oscillatory SCN network, despite their immature molecular phenotype.

In this study we provide evidence that SCN neurons maintain persistent expression of paternal UBE3A protein, revealing a relaxation of *Ube3a* imprinting in the SCN that is not typical of most neurons. It remains to be determined whether this feature of murine *Ube3a* expression is conserved in the human brain. Nevertheless, the identification of novel sites in the brain in which *Ube3a* imprinting is differentially regulated has broad implications. The most promising therapeutic strategies for AS take advantage of unsilencing of the paternal *UBE3A* allele^46^,^47^; neurons that exhibit differential parental *Ube3a* expression bias, such as those of the SCN, may allow inroads toward further understanding of the molecular mechanisms that regulate *Ube3a* imprinting, and thereby, the refinement of AS therapeutics.

## Methods

### Animals, Tissue Preparation and Immunofluorescence

All animal procedures were approved by the Institutional Animal Care and Use Committee of the University of North Carolina School of Medicine and were performed in accordance with the guidelines of the U.S. National Institutes of Health. AS model^19^ (*Ube3a*^*m*–/*p*+^) (Jackson Labs Stock # 016590; C57BL6) mice and their wildtype littermates were generated by breeding *Ube3a*^*m*+/*p*–^ females with wildtype males. Experimental litters that included *Ube3a*^*m*–/*p*–^ mice were generated by breeding *Ube3a*^*m*+/*p*–^ females with *Ube3a*^*m*+/*p*–^ males. Experimental trios of *Ube3a*^*YFP*^ mice^7^ (Jax Stock # 017765) were generated by breeding *Ube3a*^*m*+/*pYFP*^ females to *Ube3a*^*m*+/*pYFP*^ males, yielding *Ube3a*^*m*+/*p*+^, *Ube3a*^*mYFP*/*p*+^, and *Ube3a*^*m*+/*pYFP*^ mice. Homozygous *Ube3a*^*YFP*^ mice were excluded from experiments.

Mice were housed in 12:12 LD and given ad libitum access to food and water prior to perfusion. *Ube3a*^*YFP*^ reporter mice and their control littermates were collected at P30; all others were 2–5 months old at time of perfusion. Males and females were balanced in number across genotypes. Unless otherwise stated, mice were perfused between ZT4–ZT9. At the indicated ZTs, mice were deeply anesthetized with sodium pentobarbital (60 mg/kg, i.p.) before being transcardially perfused with room-temperature phosphate-buffered saline (PBS) immediately followed by room-temperature phosphate-buffered paraformaldehyde (pH 7.3). Optic nerves were severed prior to removing the brains from skulls, and the brains were then placed in fixative overnight at 4 °C. Fixed brains were then cryoprotected in 10%, 20%, and 30% sucrose in PBS at 4 °C for 12 hours each. Brains were then frozen in dry ice, and coronal sections (40 μm thick) were collected using a freezing sliding microtome (ThermoFisher Scientific, Kalamazoo, MI, USA). Sections were stored in a cryopreservative solution (v/v: 45% PBS, 30% ethylene glycol, 25% glycerol) at −20 °C.

Tissue sections were washed several times in PBS and blocked in PBS containing 5% normal goat serum and 0.2% Triton X-100 (NGST) for 1 hour at room temperature. Blocked tissue sections were incubated with primary antibodies diluted in NGST for 48 hours at 4 °C. Sections were then washed several times in PBS containing 0.2% Triton X-100 before incubation with secondary antibodies (diluted in NGST) for 1 hour at room temperature. The following primary antibodies were used: mouse anti-UBE3A (1:1,000, clone 3E5, Sigma Cat. #SAB1404508); mouse anti-NEUN (1:750, clone A60, Millipore, Billerica, MA, USA, Cat. #MAB377); rabbit anti-SOX2 (1:2,000, Active Motif, Carlsbad, CA, USA, Cat. #39823); rabbit anti-VIP (1:2,000, Immunostar, Hudson, WI, USA, Cat. #20077); rabbit anti-AVP (1:2,000, Millipore Cat. #AB1565); mouse anti-BMAL1 (1:2,000, Bethyl Laboratories, Montgomery, TX, USA, Cat. #A302–616 A); rabbit anti-PER2 (1:5,000, clone R38; Millipore, Cat. #AB2202); rabbit Anti-GFAP (1:750, Dako North America, Inc., Carpinteria, CA, USA, Cat. #Z0334); and chicken anti-GFP (1:2,000, Aves Labs, Tigard, OR, USA, Cat. #GFP-1020). The following secondary antibodies from Invitrogen (Carlsbad, CA, USA) were used at a 1:500 concentration: goat anti-mouse IgG_1_ Alexa Fluor 568 (Cat. #A-21124), goat anti-mouse IgG_2A_ Alexa Fluor 488 (Cat. #A-21131), goat anti-rabbit Alexa Fluor 568 (Cat. #A-11011), goat anti-rabbit Alexa Fluor 633 (Cat. #A-21071), and goat anti-chicken IgY Alexa Fluor 488 (Cat. #A-11039). Where specified, sections were incubated for 30 min following secondary antibodies with NeuroTrace 640/660 Deep-Red Fluorescent Nissl Stain (ThermoFisher Scientific, Cat. # N21483), diluted 1:200 in PBS, prior to final washes. In all experiments, 4′,6-diamidino-2-phenylindole (DAPI) was added during the secondary antibody incubation at a concentration of 700 ng/ml for nuclear counterstaining and delineation of the boundaries of the SCN. Perfusions, tissue sectioning, and immunostaining were performed by experimenters blind to genotype. Brain sections compared within figures were stained within the same experiment under identical conditions.

### Confocal Microscopy and Image Analysis

Immunostained sections containing the SCN were imaged using a Zeiss LSM 710 confocal microscope equipped with ZEN Imaging Software (Zeiss, Jena, Germany). Single-plane optical slices of 1.8 μm in thickness were acquired using a 20× objective (Plan-Apochromat 20 × /0.8 M27) at a pixel dwell time of 3.15 μsec with 4× averaging. Images containing the SCN, the 3^rd^ ventricle and surrounding hypothalamic regions, or the BNST or CeA, were acquired by tiling 3 × 3 micrographs of 1024 × 1024 pixels that were stitched using ZEN software. Images compared within figures were acquired using identical acquisition parameters. All images to be compared underwent identical manipulations for brightness and contrast, unless otherwise stated in the figure legends.

Quantification of immunofluorescent signals was performed in ImageJ^48^ (http://imagej.nih.gov/ij/). We used average intensity measurements to address whether changes in UBE3A expression altered protein levels of AVP, VIP, and BMAL1, in cell bodies as well as in neuronal processes. We also used a cell-counting approach to examine the degree of overlap of UBE3A-positive and SOX2-positive, NEUN-positive, VIP-positive, or AVP-positive cells in the SCN. To quantify total SCN signal of a given fluorophore, regions bounding the left and right SCN were created manually for each image using the DAPI channel, by an experimenter blind to genotype. The regions were applied to the channel of interest, and the mean gray values within SCN regions were measured. An oval region of a standard size across all images (150 μm × 300 μm) was also placed in the adjacent lateral hypothalamus beyond the SCN boundaries, and the average intensity within this region was subtracted from the average intensity in the SCN to account for variability in background fluorescence. Intensity measurements in the lateral hypothalamus regions (i.e., non-SCN regions) were not different across genotypes or ZTs. Expression measurements of proteins other than UBE3A, whose staining pattern made the genotype evident, were performed blind to genotype. To quantify the degree of colocalization, cell bodies positive for each immunofluorescent signal within each SCN region (defined by DAPI counterstaining) were manually counted, and the percentage of cells positive for both fluorophores was calculated. Sections containing rostral, central, and caudal regions of the SCN were imaged for all conditions and included for quantification in a balanced fashion.

### Statistical analysis

Statistical analyses were performed using GraphPad Prism version 6.00 for Windows (GraphPad Software, La Jolla, CA, USA). Unpaired two-tailed Student’s *t* tests and one-way ANOVA were used where indicated. Data are represented as mean ± s.e.m. (error bars). *P* values less than 0.05 were considered statistically significant.

## Additional Information

**How to cite this article**: Jones, K. A. *et al*. Persistent neuronal *Ube3a* expression in the suprachiasmatic nucleus of Angelman syndrome model mice. *Sci. Rep*. **6**, 28238; doi: 10.1038/srep28238 (2016).

## Supplementary Material

Supplementary Information
