# Supplementary material for: Persistent neuronal Ube3a expression in the suprachiasmatic nucleus of Angelman syndrome model mice
Source: Sci Rep. 2016 Jun 16;6:28238. doi: 10.1038/srep28238 (PMC4910164; doi:10.1038/srep28238)
Supplement: Supplementary Information [file srep28238-s1.pdf]

Persistent neuronal *Ube3a* expression in the suprachiasmatic nucleus of Angelman syndrome model mice

Kelly A. Jones[1,2], Ji Eun Han[1], Jason P. DeBruyne[3], and Benjamin D. Philpot[1,2,*]

[1]Department of Cell Biology and Physiology, UNC Neuroscience Center, and [2]Carolina Institute for Developmental Disabilities, University of North Carolina School of Medicine, Chapel Hill, NC 27599

[3]Department of Pharmacology & Toxicology, Neuroscience Institute, Morehouse School of Medicine, Atlanta, GA 30310

*denotes corresponding author

Corresponding author:

Ben Philpot
115 Mason Farm Rd., Campus Box 7545
Chapel Hill, NC 27599
Phone: (919) 966-0025, Fax: (919) 966-3870
bphilpot@med.unc.edu

Supplementary Figures S1-S4

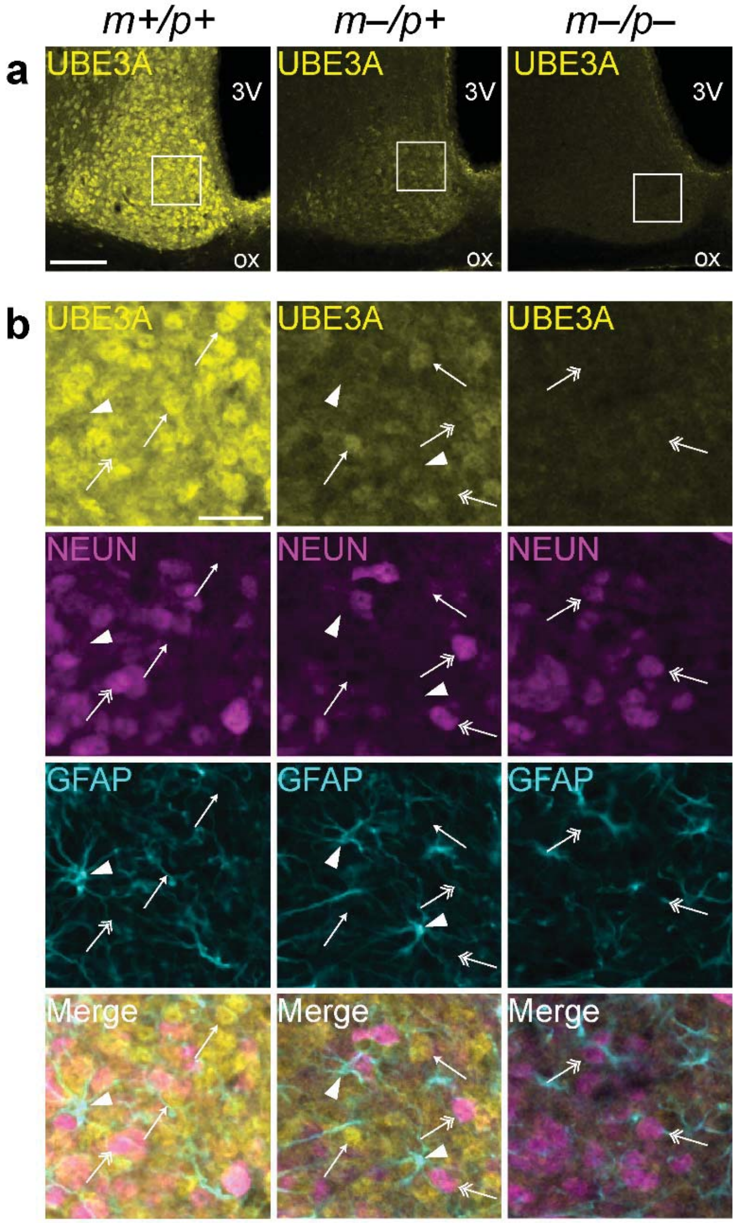

Figure S1: UBE3A, NEUN, and GFAP colocalization in the SCN.

(**a**-**b**) Low-magnification (**a**) and high-magnification (**b**) images of UBE3A, NEUN, and GFAP immunofluorescence in the SCN of *Ube3a*$^{m+/p+}$, *Ube3a*$^{m-/p+}$, and *Ube3a*$^{m-/p-}$ mice. White boxes in **a** indicate digitally zoomed regions in **b**. UBE3A signal is detectable in the SCN of *Ube3a*$^{m-/p+}$ mice, but is not present in *Ube3a*$^{m-/p-}$ mice. UBE3A signal in *Ube3a*$^{m-/p+}$ SCN only partially colocalizes with NEUN staining; arrows indicate UBE3A-positive cells, which are largely distinct from NEUN-positive cells (double-headed arrows). GFAP-positive astrocytes express very low levels of paternal UBE3A in *Ube3a*$^{m-/p+}$ SCN (arrowheads). *3V*, third ventricle; *ox*, optic chiasm. Scale bar, 100 µm (**a**); 25 µm (**b**).

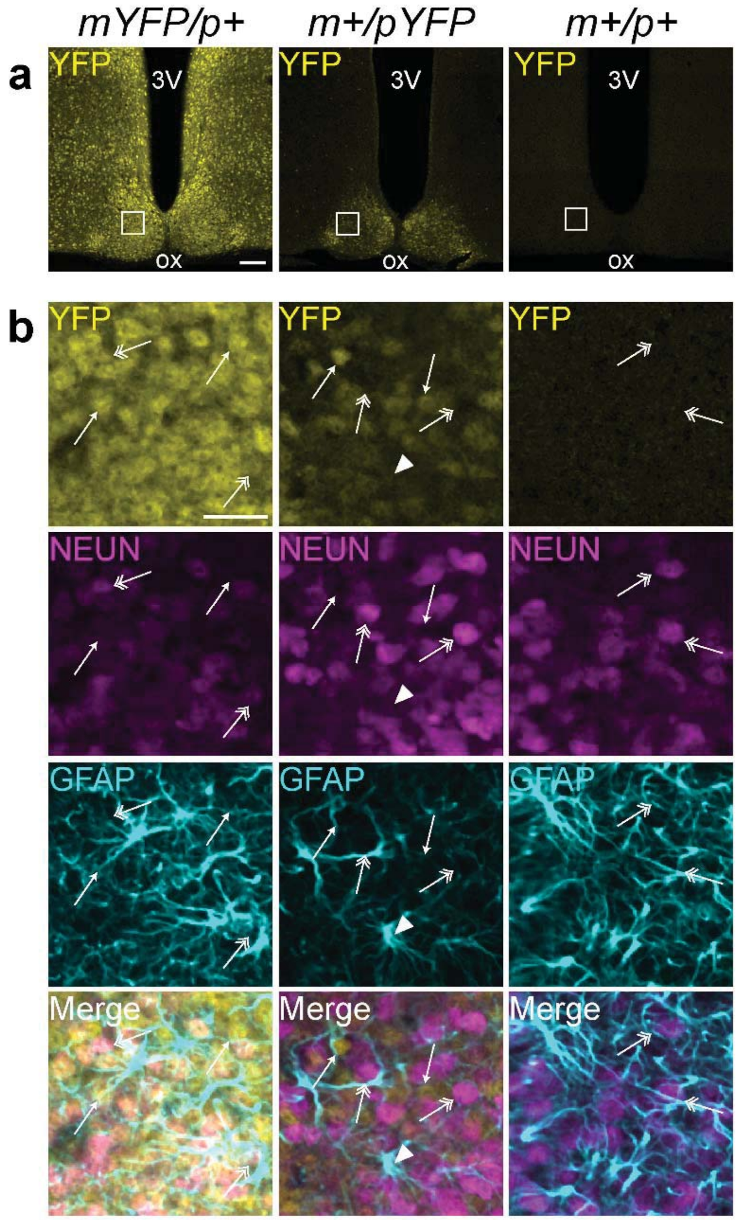

Figure S2: UBE3A-YFP, NEUN, and GFAP expression patterns in the SCN.

(**a**-**b**) Low-magnification (**a**) and high-magnification (**b**) images of YFP, NEUN, and GFAP immunofluorescence in coronal sections through the SCN from *Ube3a*$^{mYFP/p+}$, *Ube3a*$^{m+/pYFP}$, and *Ube3a*$^{m+/p+}$ mice. White boxes in **a** indicate digitally zoomed regions in **b**. UBE3A-YFP signal was amplified by immunostaining with a GFP antibody. Paternal YFP expression is detectable in a subset of SCN neurons, while maternal YFP signal is ubiquitous. *Ube3a*$^{m+/p+}$ sections are negative for YFP signal. Arrows indicate YFP-positive cells, which are largely distinct from NEUN-positive cells (double-headed arrows). GFAP-positive astrocytes express very low levels of paternal UBE3A in *Ube3a*$^{m–/p+}$ SCN (arrowheads). *3V*, third ventricle; *ox*, optic chiasm. Scale bars, 100 μm (**a**); 25 μm (**b**).

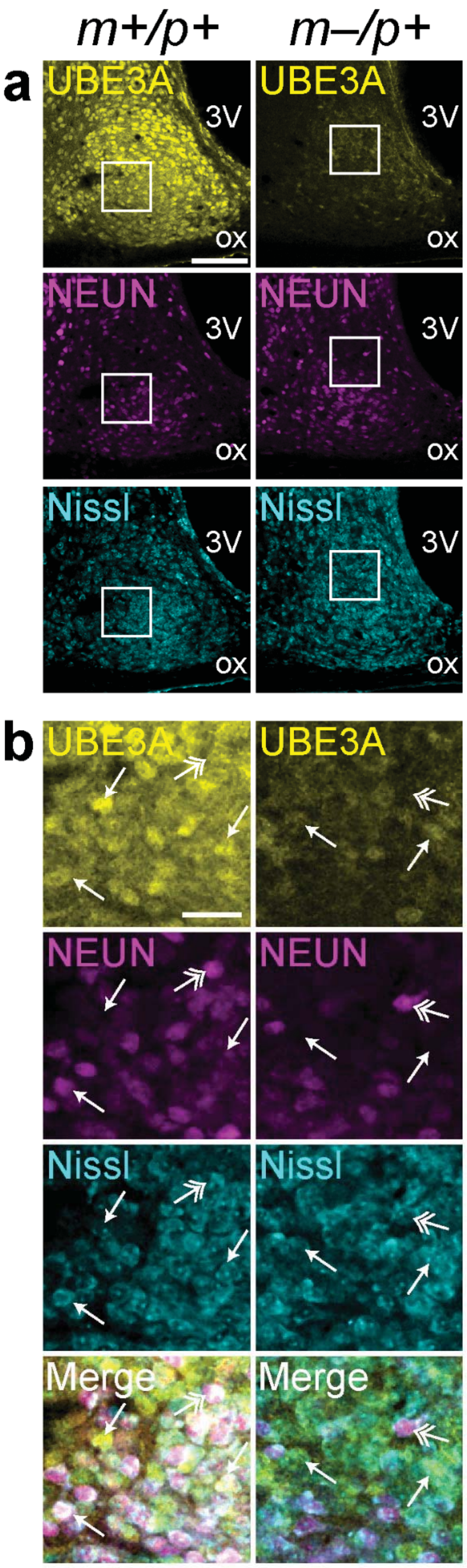

Figure S3: UBE3A, NEUN, and Nissl immunofluorescence in the SCN.

(**a**-**b**) Low-magnification (**a**) and high-magnification (**b**) images of UBE3A, NEUN, and NeuroTrace Nissl immunofluorescence in coronal sections through the SCN from *Ube3a*$^{m+/p+}$ and *Ube3a*$^{m–/p+}$ mice. White boxes in **a** indicate digitally zoomed regions in **b**. In *Ube3a*$^{m–/p+}$ sections, 90.1 +/- 0.776 % of Ube3a-positive cells were also positive for Nissl stain (n = 3 mice, 2-3 sections per mouse, 88-176 cells per section). Arrows indicate UBE3A-positive cells that are also positive for Nissl; double-headed arrows indicate NEUN-positive cells, which are largely distinct from UBE3A-positive cells. *3V*, third ventricle; *ox*, optic chiasm. Scale bars, 100 µm (**a**); 25 µm (**b**).

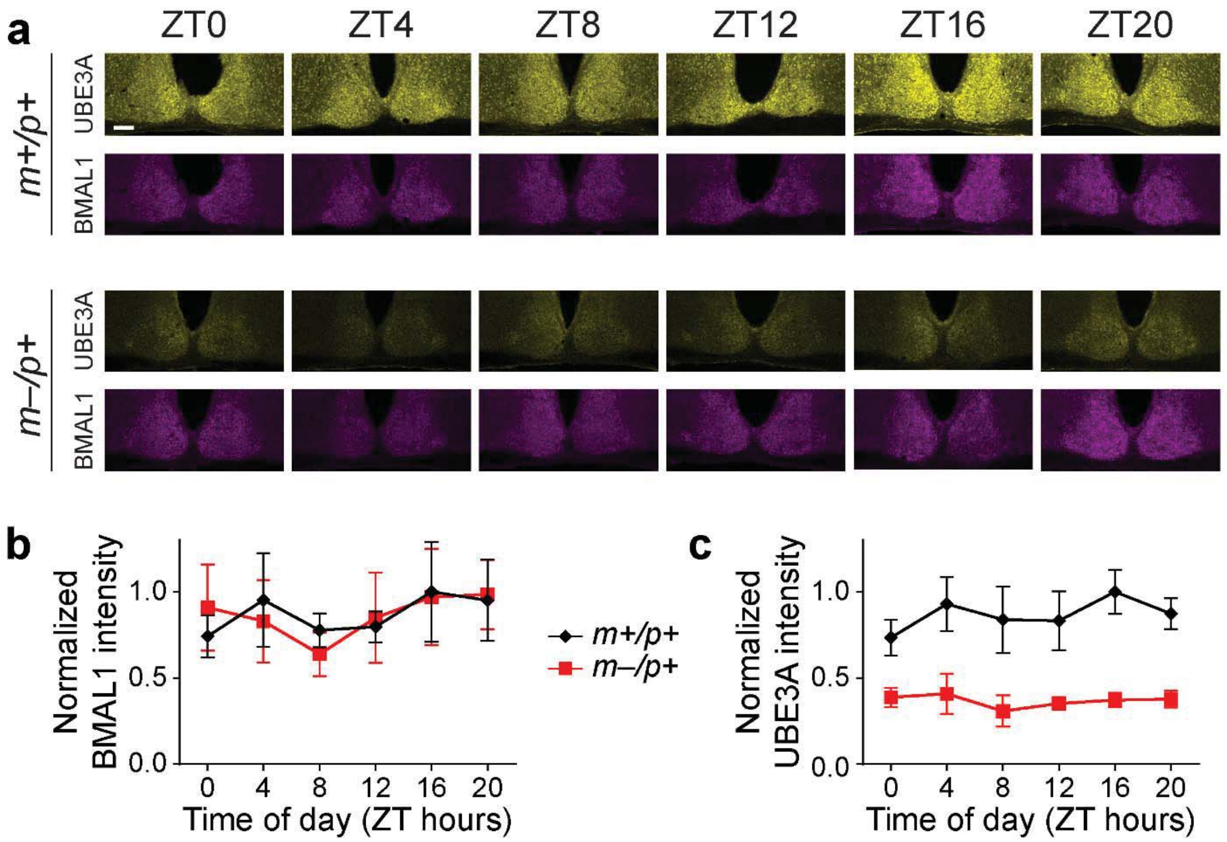

Figure S4: Expression of BMAL1 across the day is unchanged in AS model mice. (**a**) BMAL1 and UBE3A expression in the SCN of *Ube3a*$^{m+/p+}$ and *Ube3a*$^{m-/p+}$ mice sacrificed at the indicated *zeitgeber* times (ZT) across the day. (**b**) Quantification of BMAL1 immunofluorescent signal in the SCN across the day, normalized to *Ube3a*$^{m+/p+}$, ZT16 (two-way ANOVA: main effect of ZT, $F_{(5,36)} = 0.4495$, $p = 0.8108$; main effect of genotype, $F_{(1,36)} = 0.002285$, $p = 0.9621$; interaction, $F_{(5,36)} = 0.1396$, $p = 0.9819$; n = 4 mice per ZT and genotype). (**c**) Quantification of UBE3A immunofluorescent signal in the SCN across the day, normalized to *Ube3a*$^{m+/p+}$, ZT16 (two-way ANOVA: main effect of ZT: $F_{(5,36)} = 0.4026$, $p = 0.8437$; main effect of genotype: $F_{(1,36)} = 57.64$, $p < 0.0001$; interaction, $F_{(5,36)} = 0.3181$, $p = 0.8988$; n = 4 per ZT and genotype). Scale bar, 100 µm.
